# Spatial and Temporal Patterns of Carbon Storage in Forest Ecosystems on Hainan Island, Southern China

**DOI:** 10.1371/journal.pone.0108163

**Published:** 2014-09-17

**Authors:** Hai Ren, Linjun Li, Qiang Liu, Xu Wang, Yide Li, Dafeng Hui, Shuguang Jian, Jun Wang, Huai Yang, Hongfang Lu, Guoyi Zhou, Xuli Tang, Qianmei Zhang, Dong Wang, Lianlian Yuan, Xubing Chen

**Affiliations:** 1 Key Laboratory of Vegetation Restoration and Management of Degraded Ecosystems, South China Botanical Garden, Chinese Academy of Sciences, Guangzhou, China; 2 College of Life Science, Hainan Normal University, Haikou, China; 3 College of Environment and Plant Protection, Hainan University, Haikou, China; 4 Research Institute of Tropical Forestry, Chinese Academy of Forestry, Guangzhou, China; 5 Department of Biological Sciences, Tennessee State University, Nashville, TN, United States of America; 6 School of Life Sciences, Central China Normal University, Wuhan, China; Wuhan Botanical Garden, Chinese Academy of Sciences, Wuhan, China, China

## Abstract

Spatial and temporal patterns of carbon (C) storage in forest ecosystems significantly affect the terrestrial C budget, but such patterns are unclear in the forests in Hainan Province, the largest tropical island in China. Here, we estimated the spatial and temporal patterns of C storage from 1993–2008 in Hainan's forest ecosystems by combining our measured data with four consecutive national forest inventories data. Forest coverage increased from 20.7% in the 1950s to 56.4% in the 2010s. The average C density of 163.7 Mg C/ha in Hainan's forest ecosystems in this study was slightly higher than that of China**'**s mainland forests, but was remarkably lower than that in the tropical forests worldwide. Total forest ecosystem C storage in Hainan increased from 109.51 Tg in 1993 to 279.17 Tg in 2008. Soil C accounted for more than 70% of total forest ecosystem C. The spatial distribution of forest C storage in Hainan was uneven, reflecting differences in land use change and forest management. The potential carbon sequestration of forest ecosystems was 77.3 Tg C if all forested lands were restored to natural tropical forests. To increase the C sequestration potential on Hainan Island, future forest management should focus on the conservation of natural forests, selection of tree species, planting of understory species, and implementation of sustainable practices.

## Introduction

Carbon (C) storage in forest ecosystems is one of the largest and most active components of C cycling in terrestrial ecosystems and plays an important role in global C cycling and climate change [1], [2]. Information on the spatial distribution of C sources and sinks and their temporal changes is critical for understanding C cycle mechanisms and is essential for formulating climate change policies [3]. As a result, estimation of C budgets at large spatial scales has received increasing attention in recent years [4].

While occupying only 6% of land area, tropical forests contain about 40% of the stored C in the terrestrial biosphere, with vegetation accounting for 58% and soil accounting for 41% [5]. However, there is substantial uncertainty about the estimates of C storage. Conflicting results on tropical forest C storage have been reported. Houghton et al. (1992), for example, indicated that tropical forests are a C source (from 1.2 to 2.2 Pg C/yr) because of deforestation and forest degradation [6]. Malhi and Grace (2000), in contrast, reported that tropical forests are C sinks (1–3 Pg C/yr) while northern forests are C sources [7]. Further studies on C storage in tropical forests at large scales are still needed.

Hainan, the largest tropical island and the second largest island province in China, is part of the Indo-Burma biodiversity hotspot and harbors large areas of tropical forests. Several studies have been conducted on forest resources and C storage on Hainan Island, but produced remarkably varying results. For example, Fang et al. (1996) reported that the total biomass of forests on Hainan Island was 59.79 Tg during 1984–1988 [8]. Zhao and Zhou (2004) found that the forest C storage on the island was 30.92 Tg during 1989–1993 [9]. After considering forest age and vegetation types, Wang (2001) reported that the forest C storage was only 23.21 Tg [10]. Cao et al. (2002) reported that forest C stored in vegetation increased from 30.45 Tg in 1979 to 37.74 Tg in 1993 [11]. Li and Lei (2010) estimated that the total C storage was as high as 50.83 Tg in 2004–2008, while Guo et al. (2013) recently reported the total forest C storage was 37.3 Tg [12], [13].

The large discrepancies among those studies are probably due to differences in the methods used to calculate C storage. While all studies used the data from national forestry inventories (seven inventories have been conducted since 1973) conducted by the Sate Forest Agency on Hainan Island, the studies used different inventory datasets, different components of C storage, C concentration coefficients (i.e. the proportion of carbon contained in dry mass of plant organs), or age structures. For example, Cao et al. (2002), used a C concentration coefficient of 0.50 while Wang et al. (2001) used a coefficient of 0.45 [11], [14]. Although C storage in ecosystems includes both biomass C and soil C, all of the previous studies considered only the C stored in tree vegetation and failed to consider that stored in the understory or soil. In addition, the spatial distribution of C storage on Hainan Island has not been reported. Thus, it remains unclear how the spatial and temporal patterns of C storage have changed in forest ecosystems during 1993–2008 on Hainan Island, Southern China.

The goal of this study was to examine the spatial and temporal patterns of C storage in forest ecosystems on Hainan Island, China. The specific objectives were to determine: 1) changes in C density of forest vegetation on Hainan Island from 1993–2008; 2) the temporal and spatial patterns of C storage in forest ecosystems on Hainan Island during this period; and 3) how the potential for C storage can be increased.

## Materials and Methods

### Ethics Statement

This study was based on forest inventory data and our field measurements. For the field study, all necessary permits were obtained from Hainan Bureau of Forestry. The field study did not involve endangered or protected species.

### Description of Hainan Island

Hainan Island has a land area of 33,920 km^2^ and is located at the northern edge of the tropics (latitude 18°10′–20°10′N, longitude 108°37′–111°03′E). Its tropical monsoon climate includes distinct dry and wet seasons and typhoons. Average annual rainfall is 1500–2500 mm, and average annual temperature is 22–26°C. The soil type is mainly laterite. The main zonal vegetation types include tropical rain forest and tropical mountain rain forest. The island contains more than 4200 plant species (259 families, 100 genera) including about 2000 tropical species [15].

### Vegetation classification based on remote sensing and image processing

We collected the Landsat TM satellite images (November 2008), 1∶250,000 Digital Elevation Model (DEM), Hainan forest maps (1∶500,000), and administrative maps. The images were processed using ERDAS IMAGINE 8.31 [4]. This included geometric correction processing, unsupervised classification method, vegetation information extraction, image classification, and determination of area statistics [16]. The image contained a total of 17 spectral clusters of land cover of which nine were vegetation. These nine spectral clusters were merged into six vegetation types based on the Chinese vegetation taxonomy system [17]: tropical natural rain forest, *Eucalyptus* plantation, rubber plantation, *Casuarina* plantation, coniferous plantation, and orchard. The spatial location of the six vegetation types was overlaid with the Hainan forest maps to show the actual geographical distribution of the studied vegetation types and created the distribution map of forests on Hainan Island in 2008. Finally, we selected the field control points to verify and correct the distribution map, and overlaid the digital map of the administrative boundary onto the processed TM image to estimate the area of each forest type [4], [18], [19].

### Forest inventory data

Forest area and timber volume for each age class and forest type have been inventoried in China once every five years since 1973 [20]. The systematic inventorying of forests on Hainan Island began in 1989 after the island became a province split from Guangdong Province. The forest inventory database used in this study included four inventories, each of which covered a 5-year period: 1989–1993, 1994–1998, 1999–2003, and 2004–2008. The inventory data included statistical report data, a plot database, and a sample trees database. The plot database contained more than 60 factors including plot number, name of dominant species, average tree diameter at breast height (DBH), average tree height, stand volume, number of standing trees (or bamboo), and litter thickness. The sample trees database contained 11 factors including the number of sampled trees, stand type, plot number, DBH, and volume. For the plot database, plots were established using a systematic sampling method. The southwest crossing point of each grid was used as a reference point to establish a 25.82-m×25.82-m plot within a 4-km×6-km grid in 1989 (1421 plots in total). Grid size was changed to 4-km×3-km in 1994 (2829 plots in total) [21].

### Field survey plots in 2012 (field sampling data)

To verify the accuracy of the forest inventory data and to estimate C storage in the understory, litter, and soil layers, we established 100 field survey plots in 2012. The plots were selected based on forest type, spatial distribution, forest area, stand volume, and age class on the island. The number of plots for each forest type was as follows: 50 for natural forest (tropical rain forest), 24 for rubber plantation, 8 for eucalyptus plantation, 3 for *Acacia* plantation, 3 for *Pinus* plantation, 2 for *Casuarina* plantation, 1 for mixed coniferous and broad-leaved species forest, 3 for mango orchard, 3 for betel nut orchard, 2 for lychee orchard, 1 for longan orchard, and 1 for other hardwood forest. There were three replicated quadrats in each plot. The area per quadrat was 3600 m^2^ for natural forest, 800 m^2^ for plantation, and 400 m^2^ for orchard. The measured variables were the same as in forest inventory. In addition, for each quadrat, we sampled plant tissue in the tree and understory layer, litter, and soil for laboratory analysis.

### Estimation of C storage in forest ecosystems

The C in forest ecosystems includes C stored in the tree layer (tree C, including tree root C), shrub layer, herb layer, litter layer, and soil layer. C storage in the tree layer was estimated by forest inventory data and validated by our field sampling data in 2012. C storage in the shrub layer, herb layer, litter layer, and soil layer in 2012 was calculated using our field sampling data. The methods for estimating C storage in these layers were described below. Since the data of C storage in the shrub layer, herb layer, litter layer, and soil layer were not included in the forest inventories, we estimated these data using the relationships between measurements of shrub, herb litter, soil layer C and tree layer C biomass measurements developed using the measurements in 2012. While C storage in shrub, herb, litter and soil layer, and tree layer C biomass varied among years, we assumed that the relationships did not change.

### Estimation of C storage in the tree layer based on forest inventory

The biomass of trees was calculated using the Biomass Expansion Factor (*f*_BEF_) method [4].
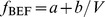
(1)where *V* is forest stand volume (*V*, m^3^ ha^−1^, for the measurement method see reference [21]), and *a* and *b* are parameters of the conversion factor of a specific tree species from volume to biomass. The conversion factor values for different dominant tree species were obtained from previous studies on Hainan Island (Table 1).

**Table 1:** The conversion formulas used in previous studies for estimating the biomass of dominant tree species on Hainan Island.

Dominant species	Biomass expansion factor (f_BEF_) formula	n*	R^2^ **	Reference
*Eucalyptus*	f_BEF_ = 0.8873+4.5539/V	20	0.80	Han et al., 2010 [22]
Rubber	f_BEF_ = 0.7975V+0.4204	18	0.87	Cao et al., 2009 [23]
*Pinus*	f_BEF_ = 0.5101V+1.0451	12	0.92	Fang et al., 2001 [24]
*Cunninghamia lanceolata*	f_BEF_ = 0.3999V+22.541	56	0.95	Fang et al., 1996, 2001 [8], [24]
Native broad-leaved species plantation (soft wood#)	f_BEF_ = 0.7564V+8.3103	12	0.91	Cao et al., 2009 [23]
Native broad-leaved species plantation (hard wood#)	f_BEF_ = 0.6255V+91.0013	19	0.86	Li, 1993 [25]
Tropical rain forest species	f_BEF_ = 1.0357V+8.0591	17	0.89	Li et al., 1995 [26]
* Acacia*	f_BEF_ = 0.6255V+91.0013	19	0.86	Zhou et al., 2008 [27]
Fruit species	f_BEF_ = 0.3154V+3.4171	6	0.76	Cao et al., 2009 [23]
*Casuarina equisetifolia*	f_BEF_ = 0.7441V+3.2377	10	0.95	Fang et al., 2001; Zhou et al., 2008 [24], [27]
Mixed coniferous and broad-leaved tree species	f_BEF_ = 0.8136V+18.4660	10	0.99	Fang et al., 2001; Zhou et al., 2008 [24], [27]

*: n is the number of trees used in developing the regression model.

**: R^2^ is the coefficient of determination. All the regression models are significant (P<0.05).

# : hard wood (wood density >0.7); soft wood (wood density <0.7).

The tree biomass at the forest stand scale (*B*, Mg ha^−1^) was calculated using the following formula:
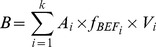
(2)where *i* is the dominant species of the forest type, *A_i_* is the forest stand area, *V_i_* is the average storage volume, and *f*_BEF*i*_ is the corresponding conversion factor of the *i* dominant species in the forest type.

The data of C storage in trees were also calculated at the city scale (Hainan Island has 18 cities. Each city represents an administration area). The biomass of the *j*-th plot in the *i*-th city (*Bij*) can be calculated using the following formula:
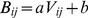
(3)where the units of *B_ij_* and *V_ij_* are Mg ha^−1^ and m^3^ ha^−1^, respectively, and *a* and *b* are conversion factors of the dominant species (Table 1).

The formula for determining the average biomass of trees in the *i*-th city (*B_i_*, Mg ha^−1^) was:
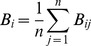
(4)where n is the total number of plots in the *i*-th city. The formula for determining the total biomass of trees in the *i*-th city (Ti) was:
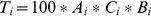
(5)where *A_i_* is the land area (unit: km^2^) in the *i*-th city, *C_i_* is the percentage of forest coverage in the *i*-th city, *B*_i_ is the average biomass of tree in the *i*-th city (Mg ha^−1^), and 100 is the unit conversion factor.

The total tree biomass in Hainan Province (*T*) was summed for all 18 county/city-level cities as:
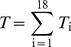
(6)


Tree C storage on Hainan Island was calculated by multiplying forest biomass (*T*) by the C concentration. The C concentration was measured in 2012.

### Estimation of C storage in the understory layer based on field sampling and laboratory analysis

The understory layer included a shrub layer (0.5 to 1.5 m tall) and a herb layer (<0.5 m tall). To estimate C storage in the understory, we collected all plant individuals including seedlings from three 5-m×5-m subquadrats in each quadrat. The collected material was dried and weighed, and 30% of the dried material per subquadrat was used for determination of C concentration by the potassium dichromate oxidation method [28]. C storage in understory layers was estimated by multiplying the dry mass of the ground layer collected from each plot and the corresponding ground layer C concentration [29].

### Estimation of C storage in the litter layer based on field sampling and laboratory analysis

To determine litter layer C, we collected all litter from three 1-m×1-m subquadrats is each quadrat. The methods used for collection, analysis, and calculation were the same as those used for the understory.

### Estimation of C storage in the soil layer based on field sampling and laboratory analysis

For determination of C in the forest soil, we collected three soil cores (4 cm diameter and 100 cm deep) per subquadrat with a soil auger. We separated each 10-cm layer for the top 70 cm (seven layers), while the 70–100 cm depth was sampled as one layer because of its relatively constant C concentration. Soil depth varied among subquadrats, and we collected cores to the maximum depth in each case. The soil bulk density was measured in accordance with the soil layers of every 1 meter soil profile [30]. The samples were processed by the potassium dichromate oxidation method for determination of the organic matter [28].

C storage in the soil of the *j*-th plot of the *i*-th city (SOC_ij_) was calculated as:
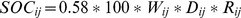
(7)where the units for SOC_ij_ are Mg ha^−1^; W_ij_ is soil bulk density (g cm^−3^); D_ij_ is soil depth (cm, soil depth ranged from 60 to 100 cm for different soil types, which depended on the soil layer depth in the field); R_ij_ is the average soil organic matter content (%) of the *j*-th plot in the *i*-th city; 0.58 is the conversion coefficient from organic matter to organic C [4]; and 100 is the unit conversion factor. The mean SOC*i* of the *i*-th city was calculated as:
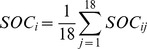
(8)where 18 represent that there are 18 cities in Hainan.

The total ecosystem C storage of *i*-th city (Total C_i_, Mg ha^−1^) was summed by vegetation C and SOC. Therefore, we used the same calculation method as above to obtain C storage data for different cities on Hainan Island.

### Mapping methods

Based on the estimation of total C storage (Vegetation C and SOC) in each city, we produced the spatial distribution map of C storage on the administration map in Hainan. The spatial distribution maps of forest ecosystem C storage on Hainan Island in 2008 were created by overlaying the spatial distribution map of tree biomass C storage in 2008 and the spatial distribution map of C storage in the shrub, herb, litter, and soil layers in 2012.

### Uncertainty analysis

There were three major sources of uncertainty in C storage estimation in forest ecosystems on Hainan Island: the uncertainty in estimation of C storage in tree layer, uncertainty in relationships used to estimate C storage in the shrub, herb, litter, and soil layers from C storage in tree layer and uncertainty in forest area estimation in our research. The uncertainty of estimations was conducted by analysis of the different error sources. The error of estimation on C storage in tree layer mainly came from the input data such as inventory of forest area and volume and model parameters associated with regression coefficients used for estimation of dominant tree biomass. The Monte-Carlo method [4] was used to calculate the uncertainty in estimation of C storage in tree layer and uncertainty in forest area estimation. The uncertainty in relationships used to calculate C storage in the shrub, herb, litter, and soil layers from C storage in tree layer in 2012 could come from two sources. One was the modeling fitting of C storage in tree layer with C storage in other layer. Another source was the application of these relationships developed in 2012 to other years. We estimated these uncertainty using error propagation method following the Guide to the Expression of Uncertainty in Measurement [31], [32]. The law of the propagation of uncertainty or the Taylor method [32] was also used in this analysis.

## Results

### Change in forest coverage from the 1940s to the 2010s and the spatial distribution of forests on Hainan in 2008

**Figure 1:**
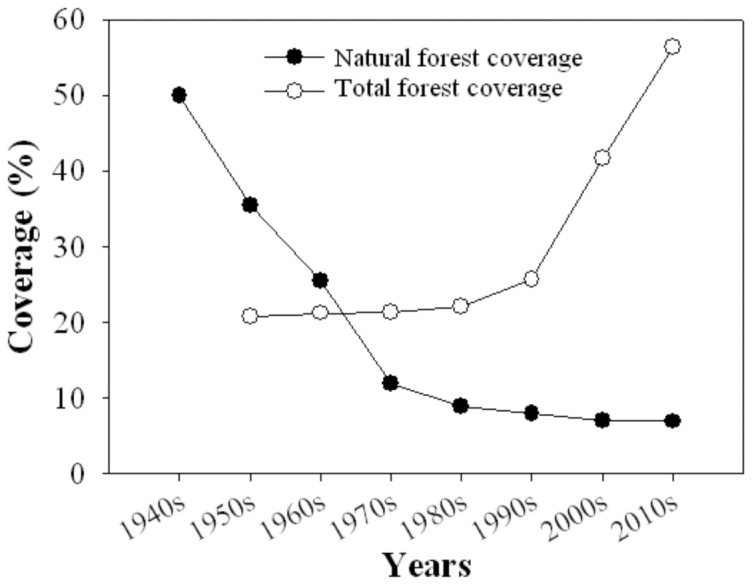
Total forest coverage and natural forest coverage on Hainan Island from 1940s to 2010s. Data are from the National Forest Resources Inventory.

Forest coverage (defined as the percentage of total land area in a region that is covered by any kind of natural or artificial forest) on Hainan Island increased from 20.7% in the 1950s to 56.4% in the 2010s. However, the natural forest coverage (defined as the percentage of total land area in a region that is covered by natural forests) decreased from 49.9% in the 1940s to 6.9% in the 2010s (Fig. 1). According to the forest inventory reports in 2008, Hainan Island had six types of forest ecosystems in 1993 and 11 types in 2008 (Table 2. Five new forest types were counted). Among them, natural tropical rain forests occupied the largest area, and followed with *Eucalyptus* (Table 2).

**Table 2:** Carbon (C) density, forest area, and C storage of forest ecosystems on Hainan Island.

Year and forest type	Area (100 ha)	C density (t/ha)						Total forest ecosystem C storage (Tg)
		Tree layer	Soil layer	Shrub layer	Herb layer	Litter layer	Forest ecosystem	
**1993**								
*Casuarina* plantation	551	12.89	116.44	2.12	0.76	1.03	133.25	7.34
Native broad-leaved species plantation (soft wood**)	179	49.59	124.06	3.30	0.52	1.13	178.60	3.20
*Eucalyptus* plantation	1596	9.51	114.79	2.04	0.82	1.01	128.16	20.45
Tropical rain forest (natural+secondary)	3539	81.58	126.99	4.84	0.43	1.17	215.01	76.09
*Pinus* plantation	144	7.56	113.56	1.99	0.86	0.99	124.96	1.80
*Cunninghamia lanceolata* plantation	48	10.83	115.49	2.07	0.80	1.02	130.21	0.62
Total	6057							109.51
**1998**								
*Casuarina* plantation	588	12.73	116.38	2.12	0.77	1.03	133.02	7.82
Native broad-leaved species plantation (soft wood**)	204	67.31	125.85	4.08	0.47	1.15	198.86	4.06
*Eucalyptus* plantation	1703	13.88	116.85	2.15	0.75	1.04	134.66	22.93
Tropical rain forest (natural+secondary)	5169	71.19	126.18	4.27	0.46	1.16	203.26	105.06
*Pinus* plantation	216	8.00	113.86	2.00	0.85	1.00	125.72	2.72
*Cunninghamia lanceolata* plantation	48	19.60	118.76	2.30	0.69	1.06	142.41	0.68
*Acacia* plantation	240	49.20	124.01	3.28	0.52	1.13	178.14	4.28
Total	8168							147.55
**2003**								
*Casuarina* plantation	516	15.06	117.30	2.18	0.74	1.04	136.31	7.03
Native broad-leaved species plantation (soft wood**)	239	57.73	124.95	3.64	0.49	1.14	187.95	4.49
*Eucalyptus* plantation	1667	14.96	117.26	2.18	0.74	1.04	136.17	22.70
Tropical rain forest (natural+secondary)	5756	69.87	126.07	4.21	0.46	1.16	201.76	116.13
*Pinus* plantation	264	15.03	117.29	2.18	0.74	1.04	136.28	3.60
*Cunninghamia lanceolata* plantation	71	20.94	119.13	2.34	0.68	1.06	144.15	1.02
Native broad-leaved species plantation (hard wood**)	23	43.97	123.36	3.08	0.54	1.12	172.07	0.40
*Acacia* plantation	384	52.77	124.42	3.43	0.51	1.13	182.26	7.00
Total	8920							162.37
**2008**								
*Casuarina* plantation	312	16.30	117.73	2.21	0.72	1.05	138.01	4.31
Native broad-leaved species plantation (soft wood**)	48	46.06	123.63	3.16	0.53	1.12	174.51	0.84
*Eucalyptus* plantation	1930	12.55	116.30	2.11	0.77	1.03	132.75	25.62
Tropical rain forest (natural+secondary)	5133	73.82	126.40	4.41	0.45	1.16	206.23	105.86
*Pinus* plantation	324	15.08	117.30	2.18	0.74	1.04	136.34	4.42
*Cunninghamia lanceolata* plantation	11	33.73	121.83	2.73	0.59	1.10	159.98	0.18
Native broad-leaved species plantation (hard wood**)	107	79.21	126.82	4.71	0.44	1.17	212.33	2.27
*Acacia* plantation	480	51.28	124.25	3.37	0.52	1.13	180.54	8.67
Mixed coniferous and broad-leaved tree species plantation.	72	50.71	124.19	3.34	0.52	1.13	179.89	1.30
Rubber plantation	5754	18.17	118.34	2.26	0.70	1.05	140.52	80.86
Orchard	3203	17.82	118.23	2.25	0.71	1.05	140.05	44.86
Total	17374*							279.17

*In 2008, rubber plantations, orchards, and mixed coniferous and broad-leaved tree species plantations were included in the statistics. The rubber plantation and orchard were first accounted, and some *Pinus* plantation developed into mixed coniferous and broad-leaved tree species plantation.

**hard wood (wood density >0.7); soft wood (wood density <0.7).

**Figure 2:**
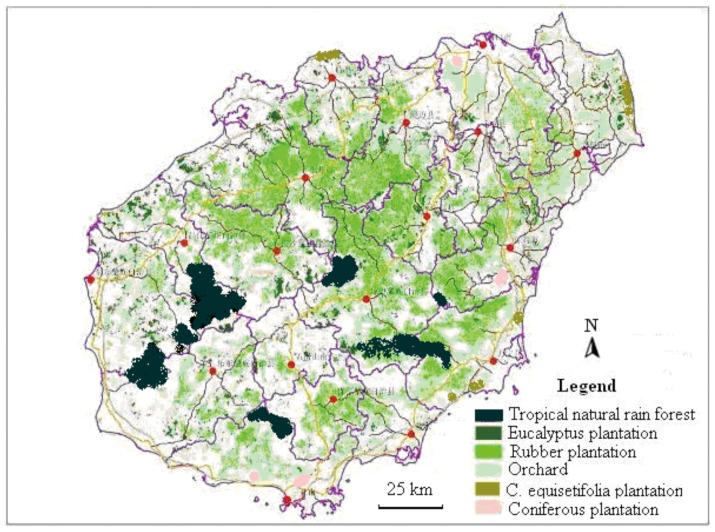
The distribution of forests on Hainan Island in 2008.

The area occupied by plantations was much larger than that occupied by natural forests in 2008 (Fig. 2). The natural tropical rain forests mainly grew in the mountainous areas of the central south of Hainan, while the plantations were distributed in the northern hilly land and the surrounding coastal plateau.

### Change in C density of the forest ecosystems from 1993 to 2008

The average C density across all forest types in Hainan in 2008 was 163.7 Mg C/ha. Among the layers of tree, shrub, herb, litter, and soil, C density in the soil layer was the largest and accounted for most of the C in each forest ecosystem (Table 2). The C density in the soil layer was 121.4 Mg C/ha, which accounted for about 74% of the total C density. The vegetation C density was about 41.2 Mg C/ha, and the C density in litter layer was only 1.1 Mg C/ha (Table 2).

The C density of most forest ecosystems on Hainan Island gradually increased from 1993 to 2008 (Table 2). The C density of forest ecosystems in 1993 varied with forest type and ranged from 125.0 Mg C/ha in *Pinus* plantations to 215.0 Mg C/ha in natural tropical rain forests. The C density of native broad-leaved species plantations (hard wood) was the highest and followed by natural tropical rain forests. In 2008, the lowest C density was in *Eucalyptus* plantations. Overall, C density was higher in natural tropical rain forests and native broad-leaved species plantations (hard wood) than in more artificial systems such as rubber and *Eucalyptus* plantations. The C storage was higher in forest types with natural regeneration (e.g., mixed coniferous and broad-leaved species plantation) than in plantations.

The average carbon density of forest ecosystems on Hainan Island increased about 2.11 Mg C/ha from 1993 to 2008 (Excluding the statistics on rubber plantations and orchards in 2008). Although the coverage of natural forest with higher carbon density decreased, the average carbon density across all forest types increased. Since other types of forests accounted for a large area and also continued to accumulating C. The results meant that the average carbon density was strongly dependent on the spatial extent of the region and types of land uses included. The average carbon density had changed along with the shifts in forest type and forested area (Table 2; Fig. 1).

### Change in C storage in forest ecosystems from 1993 to 2008

**Figure 3:**
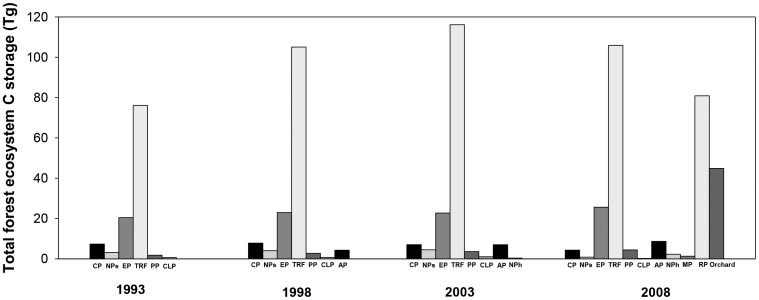
Total C storage of different forest ecosystems on Hainan Island during 1993–2008. CP: *Casuarina* plantation; NP (soft wood): Native broad-leaved species plantation (soft wood); EP: *Eucalyptus* plantation; TRF: Tropical rain forest (natural+secondary); PP: *Pinus* plantation; CLP: *Cunninghamia lanceolata* plantation; AP: *Acacia* plantation; NP (hard wood): Native broad-leaved species plantation (hard wood); MP: Mixed coniferous and broad-leaved tree species plantation; RP: Rubber plantation.

Over the past 15 years, the total forest C storage on Hainan Island gradually increased 1.55 times from 109.51 in 1993 to 279.17 in 2008. The C storage of most forest ecosystems increased from 1993 to 2009. Among them, the C storage in *Pinus* and *Eucalyptus* plantations increased 35% from 1993 to 2008. In *Casuarina* plantations and natural tropical rain forests, however, C storage increased from 1993 to 2003 but decreased from 2003 to 2008 (Fig. 3).

**Figure 4:**
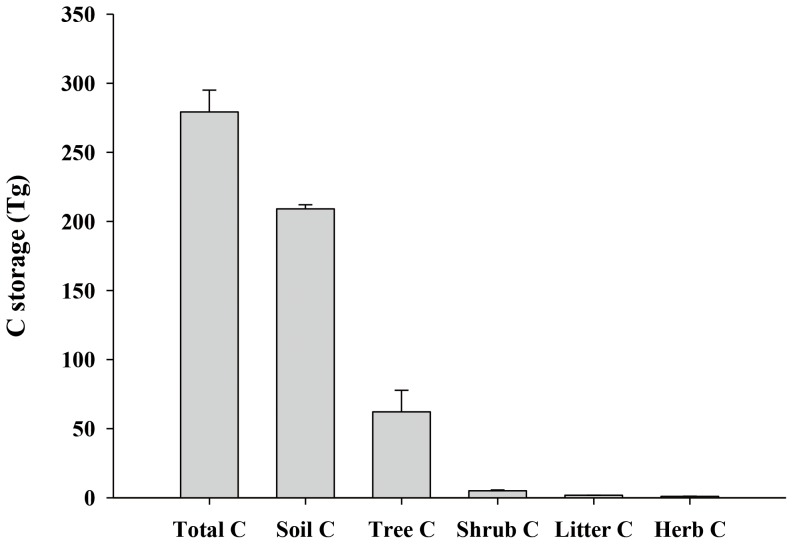
The C storage in different layers of forest ecosystems on Hainan Island in 2008. Tree C includes C in above- and below-ground biomass. Values are means ± SE.

The forest ecosystems on Hainan island in 2008 stored about 279.17 Tg C, with 209.07 Tg in the soil layer, 62.19 Tg in the tree layer, 5.06 Tg in the shrub layer, 1.06 Tg in the herb layer, and 1.79 Tg in the litter layer. Soil C accounted for 74.9% of the total C storage (Fig. 4).

### The spatial distribution of forest ecosystem C storage on Hainan Island in 2008

**Figure 5:**
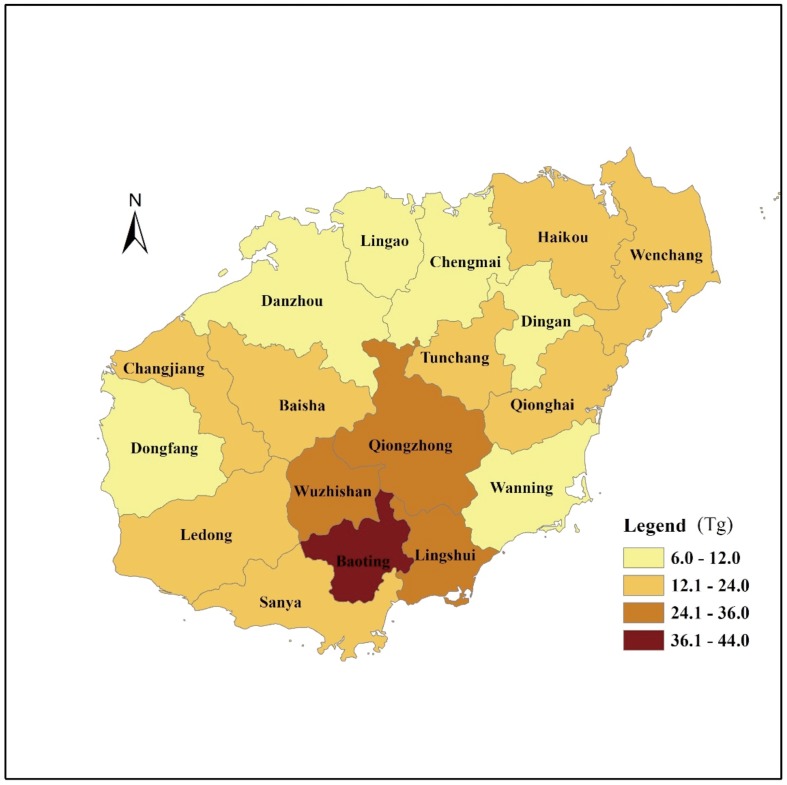
The spatial distribution of forest ecosystem C storage on Hainan Island in 2008.

The spatial distribution of forest ecosystem C storage (combination of tree biomass C storage and C storage in the understory, litter, and soil layers) in 2008 was not homogenous across the province (Fig. 5). The forest ecosystem C storage was highest in the south central region (24.1–44.0 Tg C), lowest in the north (6.0–12.0 Tg C), and intermediate in other regions (12.1–24.0 Tg C).

### Uncertainty analysis of total forest ecosystems C density

The results of uncertainty analysis of forest ecosystem C density indicated that forest ecosystem C density errors were partly came from the uncertainty in relationships used to calculate from C storage in tree layer to that in shrub, herb, litter and soil layers among three sources, it accounted for an average 3.2% (±5.05 Mg C/ha). The uncertainty on the estimation of C storage in tree layer and uncertainty in forest area estimation accounted for an average 6.1% (±10.13 Mg C/ha) of the total error (Table 3). The uncertainty of different forest types varied remarkably (Table 3).

**Table 3:** Estimations of carbon density in different layers of major forest ecosystems in 2008 with uncertainty analysis (Mean±SE).

Forest type	C density (t/ha)					
	Tree layer	Soil layer	Shrub layer	Herb layer	Litter layer	Forest ecosystem
Tropical rain forest (natural+secondary)	67.67±20.35	125.88±1.78	4.10±1.00	0.47±0.05	1.15±0.02	199.27±20.45
Native broad-leaved species plantation (hard wood**)	66.34±20.19	125.76±1.80	4.03±0.98	0.47±0.05	1.15±0.02	197.75±20.30
*Acacia* plantation	54.56±8.13	124.61±0.87	3.50±0.34	0.50±0.03	1.14±0.01	184.32±8.19
Native broad-leaved species plantation (soft wood**)	47.32±4.80	123.78±0.59	3.21±0.18	0.53±0.02	1.13±0.01	175.96±4.84
*Cunninghamia lanceolata* plantation	24.89±0.00	120.10±0.00	2.45±0.00	0.65±0.00	1.08±0.00	149.17±0.00
*Pinus* plantation	24.20±8.65	119.94±2.02	2.43±0.25	0.65±0.06	1.08±0.03	148.30±8.89
*Eucalyptus* plantation	18.92±9.48	118.56±2.79	2.28±0.26	0.69±0.09	1.06±0.04	141.51±9.89
*Casuarina* plantation	13.07±7.80	116.52±3.27	2.13±0.20	0.76±0.11	1.03±0.04	133.51±8.46

**hard wood (wood density >0.7); soft wood (wood density <0.7).

## Discussion

The C storage in forest ecosystems is closely related to ecosystem area and forest health. Both forest ecosystem area and health have declined on Hainan Island since 1900s [15]. The tropical forest area has decreased at a rate of 2.02% per year since 1950 [15]. The main causes of the tropical forest loss were excessive lumbering, planting of rubber trees, slash-and-burn cultivation, and unrestricted deforestation for fuels and other usages. Change from natural forests to artificial plantations has caused an obvious decrease in forest quality. Fortunately, the government realized the importance of protecting natural forests in the 1990s and prohibited further deforestation on Hainan Island. Rubber plantations, *Eucalyptus* plantations, and orchards, however, remain abundant. Because of rapid population growth and economic development, natural tropical forests on the plains and hilly land and along the coast have mostly been destroyed, and only remain in the mountain areas. The current status reflects a long history of human disturbance and of persistent conflict between development and conservation.

The average C density of 163.7 Mg C/ha in Hainan's forest ecosystems as estimated in this study was slightly higher than the average in China's mainland forests. For example, Wang et al. (2001) and Ren et al. (2013) reported that China's mainland forests contain a total of 141.3–147.5 Mg C/ha, with an average of 36–42 Mg C/ha in the vegetation and 105.3 Mg C/ha in the soil [4], [14]. Among those forest types in Hainan in 2008, the average C density of natural forest was the highest and was 206.23 Mg/ha in total, 78.68 Mg/ha in vegetation layer, 1.16 Mg/ha in litter layer and 126.40 Mg/ha in soil layer. The average C density of other plantation forests varied from 132.75 to 180.54 Mg C/ha. Hainan could not provide more land for planting trees [21], therefore, the potential carbon sequestration scenario was that all forested lands were restored to natural tropical forests. The potential carbon sequestration would be 77.3 Tg C. In addition, our estimation was lower than the C density in tropical forests worldwide (279 Mg/ha in total, 157 Mg/ha in vegetation and 122 Mg/ha in soil) without considering the influence of climate, fertility and other limiting factors to the forest growth [2]. The C density in forest soils on Hainan Island was close to the average C density in soil of tropical forests worldwide, but the C density in the vegetation layer in forests on the island was far less than that in tropical forests worldwide. The C storage on Hainan Island could be increased by selecting tree species with high C densities and by improving community structure. The C density in the forest ecosystems on Hainan Island was high in the soil and vegetation layers and was low in the litter layer. The low C density in the litter was reasonable because litter decomposition and nutrient cycling should occur at rapid rates under the high temperate and moisture conditions on Hainan Island.

The total forest ecosystem C storage on Hainan Island increased from 109.51 Tg in 1993 to 279.17 Tg C in 2008, with a total increase of 169.66 Tg. The increase was partially due to a 30% net increase in forest coverage during this period (Fig. 1) and partially due to the shifts in forest types. This increase was similar to the average increase in forest ecosystems in China [24]. It is worth noting that, if the C stored in rubber plantations and orchards was removed from the calculation, the total forest C storage on Hainan Island would increase by only 43.94 Tg. Another finding was that C storage from 2003 to 2008 did not increase much or even decreased slightly. This occurred because local farmers planted large areas with rubber plantations, *Eucalyptus* plantations, and orchards for economic reasons. The local government encouraged farmers to convert the existing commercial forest stand such as rubber plantations, *Eucalyptus* plantations, and orchards into ecological forest (i.e. the forests or plantations to provide ecosystem services and social services in important eco-regions or fragile regions). However, the annual compensation fee of ecological forests was only about 25% of the commodity value of plantation such as timber, rubber, and fruits [34], [35].

Rubber plantations, pulp plantations (*Eucalyptus* and *Acacia*), and orchards represent a serious threat to Hainan's natural tropical forests and C storage. The regrowth of tropical secondary forests and plantations cannot offset the C that is released as a consequence of forest deforestation, resulting in an overall net C loss on tropical lands. The C density varied among different forest types, and the C density of natural tropical rain forest was higher than that of other forest types on Hainan Island. Although Song et al. (2014) hypothesized that rubber plantations in tropical China may act as a large C sink, they were not a C sink when the deforestation of pre-existing tropical forests was considered during the establishment of rubber plantations [33]. Our previous study of C storage in *Eucalyptus* plantations and orchard on mainland China showed similar results as the rubber plantations [4]. Those studies indicated that the conversion from natural forest to plantation would result in decreasing C storage. However, farmers preferred to cut natural forests, grow the fast growing commercial trees, and sell timber to obtain the immediate economic benefits. They seldom considered the tradeoffs between conservation and agriculture [15]. Conversion of remaining natural forests to plantations would result in a loss of 105 Tg C, thus preservation of remaining natural tropical forests could make an important contribution to carbon sequestration and other ecosystem services on the island. Therefore, we provide the following recommendations to increase C sequestration in forest ecosystems: protection of all natural forests, afforestation in barren lands or waste lands, planting hard wood native species with high C fixation abilities, and restoration of forests from croplands in low productivity areas.

The estimation of forest C storage on Hainan Island varied among studies due to that various methods were used by investigators and the forest ecosystems are complex in nature. To guide climate change studies, Intergovernmental Panel on Climate Change (IPCC) published a methodological and technological guideline [36]. We applied a method that similar to the method recommended by the IPCC. Compared to other methods in previous studies [8]–[12], our estimation accounted for additional C storage in the understory layer, litter layer and soil layer and directly measured C concentration coefficient of plant organs. In addition, the uncertainty analysis of ecosystem C density, and the temporal and spatial heterogeneity of C storage were first studied, which provided more useful information for forest management in Hainan.

## Conclusions

By combining field measurements with data from forest inventories, we quantified the C storage in tropical forest ecosystems on Hainan Island between 1993 and 2008. The average C density in Hainan's forests in 2008 was 163.7 Mg C/ha, with 121.4 Mg C/ha in the soil, 1.1 Mg C/ha in the litter, and 41.2 Mg C/ha in the vegetation (trees, shrubs, herbs, including their roots). The C density of Hainan's forests was higher than the average C density of terrestrial forest ecosystems in China but lower than the worldwide average for such ecosystems. Hainan's tropical forest ecosystems stored 109.51, 147.55, 162.37, and 279.17 Tg C in total in 1993, 1998, 2003, and 2008, respectively. The total C storage in the above- and below-ground portions of forest ecosystems increased over time because of the increase in forest area and the forest type change. The spatial distribution of forest C storage on Hainan Island has been and remains uneven, and the spatial heterogeneity is related to land use, forest type, soil type and climate factors. With the increase in forest area and forest development on Hainan Island, C storage is expected to continuously increase. The potential carbon sequestration was 77.3 Tg C if all the forest stands were still natural tropical forests in 2008. From C sequestration point of view, future forest management should focus on the selection of tree species, the rational planting of understory vegetation at plantations, and implementation of sustainable practices.

## References

[1] Ciais P (1995) A large northern hemisphere terrestrial CO_2_ sink indicated by the ^13^C/^12^C ratio of atmospheric CO_2_ . Science 269: 1098–1102. 17755534 10.1126/science.269.5227.1098

[2] Lal R (2005) Forest soils and carbon sequestration. For Ecol Manag 220: 242–258.

[3] Houghton RA (2005) Aboveground forest biomass and the global C balance. Glob Change Biol 11: 945–958.

[4] Ren H, Chen H, Li L, Li P, Hou C, et al (2013) Spatial and temporal patterns of carbon storage from 1992 to 2002 in forest ecosystems in Guangdong, Southern China. Plant Soil 63: 123–138.

[5] Ashton MS, Tyrrell ML, Spalding D, Gentry B (2012) Managing forest carbon in a changing climate. London: Springer. 397p.

[6] Houghton RA (1992) Tropical forests and climate. Paper presented at the International workshop on ecology, conservation and management of Southeast Asian rainforests, October 12–14, Kuching, Sarawak.

[7] Malhi Y, Grace J (2000) Tropical forests and atmospheric carbon dioxide. Trends Ecol Evol 15: 332–337. 10884705 10.1016/s0169-5347(00)01906-6

[8] Fang JY, Liu GH, Xu SL (1996) Biomass and net production of forest vegetation in China. Acta Ecol Sin 16: 497–508.

[9] Zhao M, Zhou GS (2004) Carbon storage of forest vegetation and its relationship with climatic factors. Sci Geogr Sin 4: 50–54.

[10] Wang XK, Feng ZW, Ouyang ZY (2001) Vegetation carbon storage and density of forest ecosystems in China. Chinese J Appl Ecol 12: 13–16. 11813417

[11] Cao J, Zhang YL, Liu YH (2002) Changes in forest biomass carbon storage in Hainan Island over the past 20 years. Geogr Res 21: 551–560.

[12] Li HQ, Lei YC (2010) Estimation and evaluation of forest biomass carbon storage in China. Beijing: China Forestry Publishing House. 60p.

[13] Guo ZD, Hu HF, Li P, Li NY, Fang JY (2013) Spatial-temporal changes in biomass carbon sinks in China's forests during 1977–2008. Sci China Life Sci 43: 421–431. 10.1007/s11427-013-4492-2 23722235

[14] Wang XK, Feng ZW, Ouyang ZY (2001) Vegetation carbon storage and density of forest ecosystems in China. Chinese J Appl Ecol 12: 13–16. 11813417

[15] Zhou G (1995) Influences of tropical forest changes on environmental quality in Hainan province, P.R. of China. Ecol Eng 4: 223–229.

[16] Jobin B, Beaulieu J, Grenier M, Bélanger L, Maisonneuve C, et al (2003) Landscape changes and ecological studies in agricultural regions, Québec, Canada. Landscape Ecol 18: 575–590.

[17] Hou XY (2001) The vegetation atlas of China. Beijing: Science Press. 280p.

[18] Achard F, Eva H, Mayaux P (2001) Tropical forest mapping from coarse spatial resolution satellite data: production and accuracy assessment issues. Int J Remote Sens 22: 2741–2762.

[19] Lee TM, Yeh HC (2009) Applying remote sensing techniques to monitor shifting estuary mangrove communities, Taiwan. Ecol Eng 35: 487–496.

[20] Guo Z, Fang J, Pan Y, Birdsey R (2010) Inventory-based estimates of forest biomass carbon stocks in China: A comparison of three methods. For Ecol Manag 259: 1225–1231.

[21] Hainan Bureau of Forestry (1999) Forest resource statistics of China (1994–1998). Department of Forest Resource and Management, Hainan Bureau of Forestry, Haikou, China.

[22] Han FY, Zhou QY, Chen SX, Chen WP, Li TH, et al (2010) Study on biomass and energy of two different-aged Eucalyptus stands. Forest Res 23: 690–696.

[23] Cao JH, Jiang JS, Lin WF, Xie GS, Tao ZL (2009) Biomass of *Hevea* clone PR107. Chinese J Trop Agr 29: 1–8.

[24] Fang J, Chen A, Peng C, Zhao S, Ci L (2001) Changes in forest biomass carbon storage in China between 1949 and 1998. Science 292: 2320–2322. 11423660 10.1126/science.1058629

[25] Li YD (1993) Comparative analysis for biomass measurement of tropical mountain rain forest in Hainan Island, China. Acta Ecol Sin 13: 314–320.

[26] Li YD, Wu ZM, Zeng QB, Zhou GY, Chen BF, et al (1998) Carbon pool and carbon dioxide dynamics of tropical mountain rain forest ecosystem at Jianfengling, Hainan Island. Acta Ecol Sin 18: 371–378.

[27] Zhou C, Wei X, Zhou G, Yan J, Wang X, et al (2008) Impacts of a large-scale reforestation program on C storage dynamics in Guangdong, China. For Ecol Manag 255: 847–854.

[28] Liu GS, Jiang NH, Zhang LD, Liu ZL (1996) Soil physical and chemical analysis and description of soil profiles. Beijing: Standards Press of China. 266p.

[29] Liu H, Ren H, Hui D, Wang W, Liao B, et al (2014) Carbon stocks and potential carbon storage in the mangrove forests of China. J Environ Manag 133: 86–93. 10.1016/j.jenvman.2013.11.037 24374165

[30] Zhang JP, Shen CD, Ren H, Wang J, Han WD (2011) Estimating change in sedimentary organic carbon content during mangrove restoration in Southern China using carbon isotopic measurements. Pedosphere 22: 58–66.

[31] Cox M, Harris P, Siebert BPL (2003) Evaluation of measurement uncertainty based on the propagation of distributions using Monte Carlo simulation. Meas Tech 46: 824–833.

[32] Krouwer JS (2003) Critique of the guide to the expression of uncertainty in measurement method of estimating and reporting uncertainty in diagnostic assays. Clin Chem 49: 1818–1821. 14578312 10.1373/clinchem.2003.019505

[33] Song QH, Tan ZH, Zhang YP, Sha LQ, Deng XB, et al (2014) Do the rubber plantations in tropical China act as large carbon sinks? Forest 7: 42–47.

[34] Deng F, Chen Q, Chen X (2007) Comparison of ecological service among natural forest, rubber and Eucalyptus plantations. J South China Univer of Trop Agr 13: 19–23.

[35] Ren H, Shen W, Lu H, Wen X, Jian S (2007) Degraded ecosystems in China: Status, causes, and restoration efforts. Landscap Eco Engine 3: 1–13.

[36] IPCC (2000) Land use, land-use change, and forestry. Cambridge: Cambridge University Press. 375p.

